# Family Connections in different settings and intensities for underserved and geographically isolated families: a non-randomised comparison study

**DOI:** 10.1186/s40479-019-0111-6

**Published:** 2019-08-26

**Authors:** Sophie I. Liljedahl, Nikolaus Kleindienst, Margit Wångby-Lundh, Lars-Gunnar Lundh, Daiva Daukantaitė, Alan E. Fruzzetti, Sofie Westling

**Affiliations:** 1 Finjagården Treatment Center, Finja, 9062, 28193 Finja, Sweden; 2 Department of Psychology, Lund University, Box 213, SE-221 00 Lund, Sweden; 3 Institute of Psychiatric and Psychosomatic Psychotherapy, Central Institute of Mental Health, Medical Faculty Mannheim, Heidelberg University, Mannheim, Germany; 4 McLean Hospital & Harvard University, 115 Mill Street, Belmont, Massachusetts USA; 5 Department of Clinical Sciences, Lund, Psychiatry, Lund University, Clinical Psychiatric Research Center, Region Skåne Lund, Sweden

**Keywords:** Family Connections, Dialectical behaviour therapy (DBT), Residential treatment, Borderline personality disorder (BPD), Enhanced service delivery, Relatives, Families

## Abstract

**Background:**

Family Connections (FC) is a multi-family skills training program for relatives of individuals with borderline personality disorder (BPD) and related difficulties, typically offered once per week for 12–14 weeks. Families with loved ones receiving residential Dialectical Behaviour Therapy DBT (DBT-R) in a different community, or those with multiple caregiving demands may have difficulty participating in weekly standard FC (FC-S). The aims of this paper are to: 1) Evaluate the results of the FC-S approach compared with an intensified weekend FC model developed for family members whose relatives are in DBT-R (FC-R); 2) Evaluate outcomes of FC-R for families with loved ones returning home from DBT-R, as little is known about how this population fares.

**Methods:**

Data were collected at pre-treatment (T1), post-treatment (T2), and at six-to-seven-month follow-up (T3) in this non-randomized comparison study. A total of 82 family members participated, 34 of whom completed the FC-S program and 48 of whom completed the FC-R program. The evaluation was based upon outcomes derived from a standard battery used in FC research, analyzed by time and treatment setting. A composite score to evaluate family distress was generated. Two-way mixed multivariate analyses of variance (MANOVA) were employed to evaluate time (pre-versus-post versus follow-up) and group (FC-S versus FC-R).

**Results:**

Scores on measures of mental health difficulties (General Severity Index), sense of burden (Burden Assessment Scale), and Global Family Functioning showed improvement over time. Having a loved one return home from DBT-R was associated with worsening on the GSI and the BAS at post-test. Notably, this deterioration was not found at follow-up.

**Conclusions:**

Little is known about families with loved ones receiving DBT-R other than the fact that their loved ones had not responded to previous services, which suggests greater complexity and chronicity. Because the family members receiving the weekend intensive FC-R version of FC demonstrated improvement, preliminary support exists for service providers to use the weekend intensive FC-R model as a time-and-cost efficient option whenever barriers exist to participating in weekly FC-S. Our findings also suggest that booster sessions may be indicated for families receiving loved ones home from DBT-R programs.

## Background

The onset of borderline personality disorder (BPD) is principally during adolescence or early adulthood, with prevalence estimates ranging from 1 to 6% of the general adult population [1, 2]. Many individuals with BPD also present with co-occurring clinical syndromes that are severe enough to impair their functioning, largely within the mood, anxiety, neuropsychiatric and other personality disorder spectra [2, 3]. For individuals with Cluster B personality disorder (including BPD) a large, national Swedish study has shown similar prevalence for women with a standardized mortality rate (SMR) of 34.5. Men with cluster B personality disorder showed a SMR of 16 for death by suicide. Death due to all causes was shown to be increased in women (SMR 6.4) and men (SMR 5.6) [4]. As stated by Lieb et al., [5] “these individuals can be distinguished from other groups by the overall degree of their multifaceted emotional pain.” (p. 453). The problems of BPD are often expressed interpersonally. Accordingly, including the family or other people close to the individual as the nearest social system within the treatment context may be more important for individuals diagnosed with BPD compared to other treatment-receiving populations [6].

### Contributors to and maintenance of BPD

The invalidating interpersonal environment is one in which the individual is ignored, misunderstood, treated indifferently or with blame not only for their distress but for the circumstances that generated the distress. In sum, invalidation communicates to the individual that they are essentially unimportant [7, 8]. Invalidating environments range from child maltreatment and, at its worst, intra-familial child sexual abuse. It can also be extra-familial, taking the form of bullying at school, on-line or at work. Invalidating environments may also characterize social or romantic relationships [9].

It is important for service providers to be mindful that the family is not necessarily the primary source of pervasive invalidation. Working from this assumption in other psychotherapeutic modalities has historically alienated families from therapists to the detriment of an important and potentially powerful therapeutic ally and support system for the person in therapy [10]. It is therefore vital to include and involve family members where consent from the individual in therapy exists and family members are available and willing [11].

### Creating a validating family environment in the context of a relative’s suicidality

A warm, safe, validating and loving family environment in which all members feel accepted and valued can be particularly challenging to establish (or maintain) when one or more individuals within the family is severely and persistently distressed to the point of recurrent suicidality. When distress is prolonged, with a tendency to escalate to include self-harming and suicidal crises, there is a risk for “burn out” of individual and family coping resources. Because of the phenomenology of BPD this risk is heightened amongst family members with a relative who has been diagnosed with BPD or a related disorder [12].

#### Family Connections

Enhancing skills to maintain a validating family environment in the face of recurrent crises, and a mandate to acknowledge and undo harmful effects of stigma within the mental health system were significant needs that *Family Connections* (FC) was created to address [13]. The FC program is a specialized multi-family skills training program developed in the United States [14]. In 2006/2007, *Socialstyrelsen*, Sweden’s national board of health and welfare, commissioned a report to evaluate FC in Sweden [15] using a manual translated to Swedish. Similar measures were included in the Swedish evaluation of FC as in the American study evaluating FC [13]. The program is aimed to support relatives of individuals receiving treatment for BPD. Subsequent FC research in Europe [16, 17] has utilized a similar battery of measures as the initial American FC studies, for consistency and cross-study comparison. We observed this convention in the current study as well.

FC in Sweden is delivered to families by therapists with DBT training [15], which is a cultural adaptation made in the Swedish context. In contrast, family members who are FC graduates deliver the FC program in Canada and the United States. There is an abundance of DBT therapists in Sweden, due to the fully socialized nature of the mental health system, whereby FC can be offered within Psychiatry for free. The program is comprised of 12–14 weekly sessions attended concurrently by all families registered for the program. There are three key goals of FC: 1) psychoeducation regarding the criteria and phenomenology of BPD, emotion dysregulation, and the transactional model for BPD; 2) learning skillful responding across a number of domains that are vital to supporting a dysregulated loved one (based both on DBT skills [18] and on DBT family skills [9]); and 3) to build a support network for participants, as they may be uniquely able to understand the strengths and needs of families with relatives diagnosed with BPD. The goals of FC and the materials to train families are published within the original FC manual [14].

Relatives in the first Swedish evaluation of FC [15] reported significant reductions in perceived burden in relation to caring for the family member in treatment, and significant improvements in mindfulness. Participants also reported significant improvements in family functioning, such as reduction in critical comments and emotional over-involvement. Family members further reported reduced perceptions of chaos and increased closeness.

Changes in the direction of improvement for families participating in FC have been reported in all FC evaluation research to date [15–17, 19]. When FC was compared with a 3-session psychoeducation group for participants in a comparison group, only FC families showed improvement [17]. That is, the psychoeducation group did not show improvement post-intervention, whereas improvement was present in the FC families’ scores as measured at post-test and at 12- and 19-month follow-up [17].

#### FC in differing delivery intensities based on treatment setting: a rationale

Almost all individuals with BPD have multiple co-principal diagnoses. Individual functioning is sometimes impaired to the point that independent living is not possible. Typically, these individuals have had numerous unsuccessful attempts at outpatient therapy and have similarly not benefitted from non-specific residential services lacking a clear therapeutic structure [20]. A private residential treatment facility in Sweden (the DBT-R setting in the current study) was developed to meet the needs of this sub-population, adapting standard DBT for use in their residential setting.

Referrals to the DBT-R setting are national. FC was adapted to be delivered over two intensive weekends split 1 month apart so family members could attend even if residing far away (FC-R). Alternatively, standard FC (FC-S) is delivered weekly in most settings. This therapeutic intensity or structure (two weekends rather than weekly for 12 weeks) was devised to reduce travel burden and time off work for relatives, both of which were predicted barriers to FC participation. The month in between weekend trainings allowed for rest, homework completion and skills practice.

#### Pioneering research testing FC outcomes

An initial evaluation of the 12-week FC-S program was conducted in the USA with 34 participating families [13]. Participants were relatives of family members with BPD and related disorders. Results indicated that relatives reported significantly greater sense of understanding how to respond to their family member when dysregulated or in crisis, reductions in sadness due to loss as well as reductions in the sense of feeling ill-equipped to manage the needs of their family member. These changes persisted at 3-month follow-up [13]. Two years later [21] a larger study replicated the findings of the initial evaluation.

If FC-R were to generate similarly positive outcomes as FC-S, this could be an important contribution to implementation of FC internationally:

1. For geographically distant family members with significant travel barriers to attending weekly FC groups
2. For communities that do not have DBT services or other evidence-based programs for people diagnosed with BPD
3. For relatives with multiple roles such as caring for elderly parents or small children, working or continuing their education in the evenings, who could not likely attend a weekly group over several months.

#### The current study

The current study is the first evaluation of FC to extend the follow-up period to six-to—7 months^1^ and to evaluate FC in two different therapeutic intensities based on treatment setting of the loved one receiving DBT. The comparison setting was a clinic offering standard outpatient DBT (DBT-S) to individuals and weekly FC-S to family members. An overarching aim of the study was to determine whether the findings in the first Swedish evaluation of FC [15] could be confirmed and extended to a situation involving implementation of the program in two different therapeutic intensities.

The six-to-seven-month follow-up period added to the current study protocol is an extension to Lundh and Wångby’s evaluation [15] as well as to the original evaluations of Family Connections in the United States [13, 21]. Six-to-seven months follow-up was selected in the current study to examine how changes in outcomes persist or desist over time and how results may vary as loved ones complete DBT-R and return home.

### Study hypotheses

1. That family members receiving either FC-S or FC-R will both report improvements post FC and at follow-up.
2. Family members who participate in FC-R may report some deterioration in outcomes when their loved one returns home from DBT-R.

## Method

### Participants

A total of *N* = 82 (M_age_ = 51.69, SD = 14.44; 57.32% female) participants completed the study during data collection that ran from 2014 to 2017. During this time a total of 11 participant cohorts completed FC (5 cohorts at the FC-S setting and 6 at the FC-R setting), ranging in group size between four to 11 participants. The sample was comprised of parents (*n* = 61, 74.4%) followed by siblings (*n* = 9, 11.0%), and others, for example loved ones in the form of closest friends (*n* = 7, 8.5%) and partners (*n* = 5, 6.1%).

### Measures

At Time 1(T1 – pre-FC) a demographic form accompanied the testing battery, with items querying age, sex, relationship, living status and degree of contact with the loved one in treatment. At Time 2 (T2 – post-FC) and Time 3 (T3–6 – 7 months following the end of FC), only questions regarding living situation and degree of contact were queried with respect to demographics.

### Brief symptom inventory

(BSI) is a short-form of the Symptom Check List (SCL-90 [22]) with a total of 53 items querying different aspects of mental health difficulties. Subscales include Anxiety, Depressiveness, Hostility, Interpersonal Sensitivity, Obsessive Compulsiveness, Phobic Anxiety, Paranoid Ideation, Psychoticism and Somatization. The scale includes a global severity index (GSI), which is calculated as the average score of the 53 items.

### Burden assessment scale

(BAS; [23]) evaluates the perceived burden of having a family member with mental health difficulties. The BAS has been translated into Swedish and validated [24]. Factor analysis confirmed three subscales: activity limitation (9 items, Cronbach’s alpha = .88), feelings of worry and guilt (5 items, Cronbach’s alpha =. 73), and social strain (5 items, Cronbach’s alpha = .75). Total scores are for the entire measure as well as the three subscales.

### Quality of life inventory

(QOLI; [25]) is a measure that queries 16 areas of life that contribute to overall well-being. Swedish norm data are generated from a random sample of adult urban dwellers [26].

### Kentucky inventory of mindfulness skills

(KIMS; [27]) is a 12-item scale comprised of four subscales. These are 1) Observe, 2), Describe, 3) Act with awareness, and 4) Accept without judgement. The KIMS has been translated and validated in Swedish [28].

### Questions about family members

(QAFM; [29]) is a 30-item measure that evaluates Expressed Emotion, a construct that was divided into sub-scales to measure relatives’ perceived criticism, relatives´ perceived emotional involvement, as well as one’s own tendency to criticize, and one’s own emotional over-engagement with respect to the family member. The QAFM has good psychometrics in both reliability and validity [29].

### The family climate scale

(FCS: [30, 31]) is comprised of a list of 85 adjectives that depict the experienced atmosphere within the family. The scale is divided into four subscales. These are closeness (alpha = .98), distance (alpha = .91), expressivity/spontaneity (alpha = .71), and chaos (alpha = .92).

### Global family functioning

In order to generate a relevant measure for the proximal aim of the intervention, that is, improvement within the family’s emotional environment as perceived by the participants, and in order to avoid multiple testing, a composite score to evaluate family distress was created. This was achieved using the Equal Weights method described by Fralicx and Raju [32]. To this end the subscales from the QAFM and from the FCS were each multiplied by the reciprocal of its standard deviation (at T1) and the resulting products were added to obtain a composite criterion [32] p.825. To facilitate the interpretation of the composite score, each subscale was centred at the unity. The selection of the subscales was done following the recommendations by Paunonen and Gardner [33] who showed that psychometric parallelism is the crucial criterion for getting valid and unbiased results from composite scores. This requires uniform intercorrelations of the underlying variables (for details see [33] p. 521–522). The following subscales have been selected to be included in the composite score for a negative family climate: Perceived Criticism, Critical Comments, Emotional Over-involvement, Expressivity / Spontaneity, and Chaos. The selection of these five subscales is supported both by unequivocally negative content of each of these subscales and by the consistently positive intercorrelations of these five. In contrast to these homogenous intercorrelations, intercorrelations with the three subscales that have not been included in the composite score (i.e. Closeness, Distance, and Perceived Emotional Involvement) were mixed. However, to exclude the possibility that the results might critically depend on the selection of subscales, a sensitivity analysis was carried out after all of the eight subscales from both the QAFM questionnaire and from the FCS had been included. Because all of the pertinent results have been fully confirmed from this sensitivity analysis and because the composite score for a negative family climate based on the five subscales is believed to be more meaningful and valid, we will report only the latter composite score.

### Procedure

#### Recruitment

In both settings, therapists informed individuals receiving DBT that the FC program was being offered by their treatment setting. If the individual receiving DBT gave consent that their family member could be approached to participate, contact information for family members was shared with therapists. Therapists then sent information regarding the FC program, information regarding this study, and consent forms to family members. Approximately 1 week after information was mailed by recommended post with pre-paid return envelopes, the therapists conducting FC called family members to ask whether they would like to participate in FC, register them if so, and to answer any questions they might have about the current study. Independence of participating in FC and the current study was emphasized, both in the information letters to prospective participants and by telephone. All study materials and procedures were formally approved by the regional ethical review board in Lund, Sweden (Dnr 2013/548).

### Changes to the research protocol

Approximately mid-way through the study, the team consulted with one of the co-developers of FC, (co-author AEF) regarding low participation rate in the T 3 follow-up assessment. It was understandable that attrition would be high given the 6-7-month post-test, in consideration for all that might be happening in the lives of participating relatives. Suggestions arising from this consultation, which were approved by the regional ethics review board (Lund: Dnr 2015/448) were to:

1. Retain data from all study-points. That is, do not consider those not participating in one time-point as drop-outs as long as they had given consent to participate at T1.
2. For the FC-R group add a booster telephone-call during the month between the final FC intensive weekend and the T2 post-test. We realized that it was possible that participants thought of FC as “ending” when they stopped participating in intensive weekends. As in DBT, homework is an essential component of treatment [12]. Adding the booster call to check homework and review material that may not have been well-understood could serve the function of extending FC for the additional month from the second half of the intensive FC weekend. T3 participation increased after introducing this change.

### Statistics

To evaluate change and direction of change in scores over time in relation to FC on the BSI, the BAS and on the composite score evaluating family distress, a two-way mixed multivariate analyses of variance (MANOVA) including one within group factor (time: pre vs post vs follow-up assessments) and one between group factor (group: FC-S versus FC-R) was conducted. Since the pre-to-post increase in contact was assessed on an ordinal scale, Spearman correlation (rho) was calculated in correlations involving pre-to-post increase in contact. *P*-values ≤0.05 (two-tailed) were considered statistically significant. Analyses were calculated using SPSS Statistics for Windows, version 24.

## Results

A total of 79.4% of the FC-S participants and 95.8% of the FC-R participants completed FC. FC-S participants could miss up to two sessions and be considered completers, whereas FC-R participants could miss zero sessions, since the entire intervention was delivered across four weekend days. For both sites, no significant differences between participants completing versus not completing FC and the post-assessments with respect to demographic variables (including sex, age) and major variables at baseline (including the GSI of the BSI, the BAS, and Global Family Functioning) were detected.

**Fig. 1:**
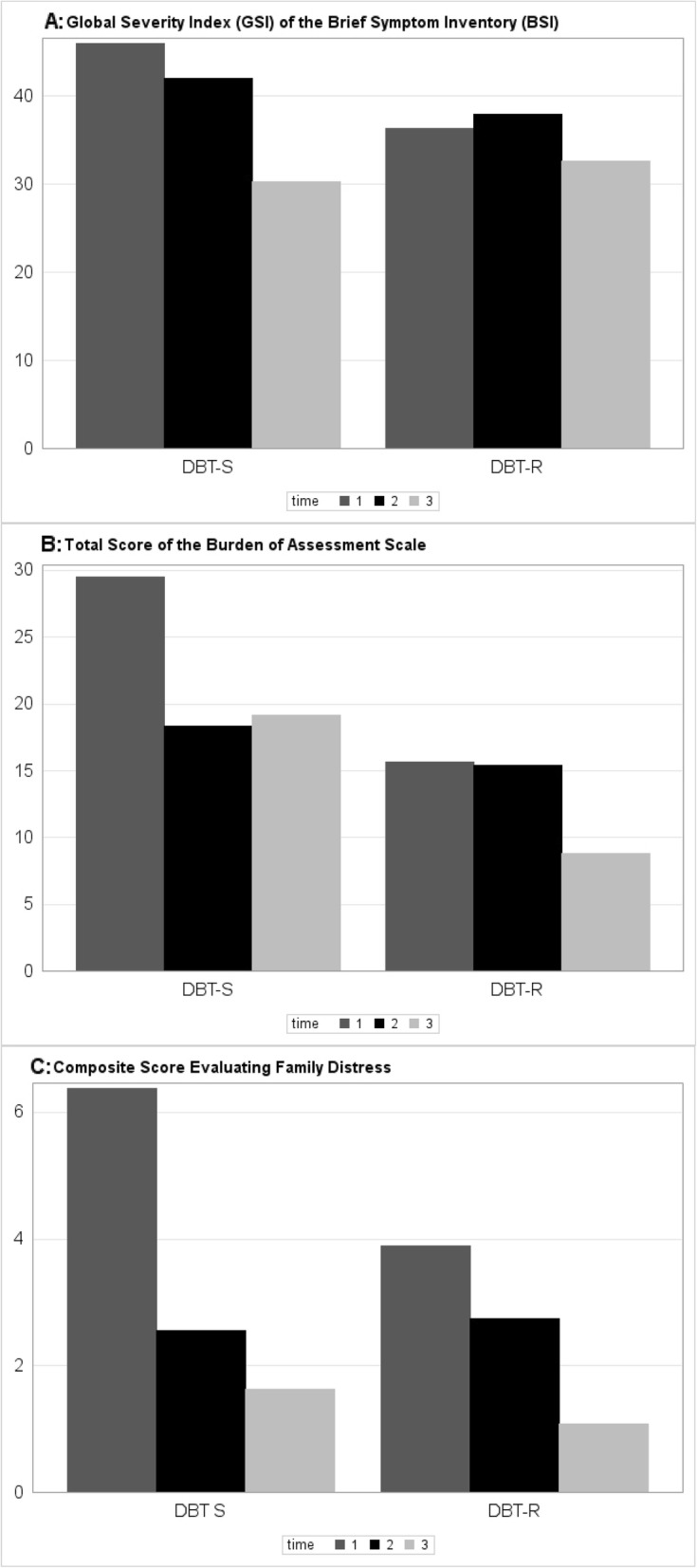
GSI, BAS, and Family Distress scores for pre (time 1), post (time 2), and follow-up (time 3) assessments

As indicated by a significant main effect of time (*F* (2,23) = 4.20, *p* = .028) in the repeated measures analyses of variance (MANOVA), FC participants improved with respect to the Global Severity Index of the BSI. The MANOVA did not yield evidence for a differential effect related to the group: Both the influence of group (*F* (1,24) = 0.123, *p* = .729) and the time*group interaction (*F* (2,23) = 1.20, *p* = .319) were not significant. Similarly, the MANOVA indicated a significant effect in relation to time (pre-FC vs post FC vs follow-up) with respect to the Burden Assessment Scale (BAS: *F* (2,17) = 5.06, *p* = .019). For the BAS both a significant group effect (*F* (1,18) = 5.26, *p* = .034) and a significant time*group interaction were detected (*F* (2,17) = 5.06, *p* = .017). As illustrated in Fig. 1b, these significant effects related to the group mainly originated from a higher level of the BAS at T1 (i.e. pre-FC) in the FC-S sample as compared to the FC-R sample. Close inspection of Fig. 1a and b further indicates that before showing improvement at the follow-up assessment, the mean levels of both the GSI and of the BAS are not improved at the post assessment in the FC-R sample.

The lack of improvement at T2 (post-test) seems to be at odds with other FC research [13, 15–17, 21]. However, if the lack of improvement was due to increased contact with the person in therapy returning home from residential treatment in the FC-R sample, it would be consistent with the second hypothesis, which was clinically driven. The return home may be accompanied by initial stress in adjusting back to life outside of a therapeutic setting both for people in therapy and their family members with whom they live. This hypothesis was tested in two post-hoc analyses relating the pre-post differences in the contact with residential participants returning home to the pre-post differences in (a) the GSI and (b) the BAS. As hypothesized, an increase in contact at post-test (T2) for the FC-R family members was significantly related to an increase in both the GSI (rho = 0.364, *p* = 0.048) and the BAS (rho = 0.459, *p* = 0.021).

As indicated by a further post-hoc analyses carried out with the GSI and with the BAS, the subscale on Worry and Guilt was particularly responsive to the FC intervention (*F* (2,21) = 11.79, *p* = <.001) (for details see Table 1). With respect to the most relevant proximal measure of family functioning, that is, the Global Family Functioning composite score evaluating family distress, a significant effect emerged for time from the MANOVA (*F* (2,17) = 9.73, *p* = .002). As illustrated in Fig. 1c this effect was consistently found in both sites and was extended to the six-to-seven-month follow-up assessment. The difference between groups in family distress was not significant (*F* (1,18) = 0.836, *p* = .373) and there was no significant time*group interaction (*F* (2,17) = 3.39, *p* = .058). Post-hoc analyses regarding the original subscales from the Questions About the Family questionnaire and from the Family Climate Scale confirmed that the effect of FC comprises the subscales pertinent to the Global Family Functioning composite score evaluating family distress (e.g., the subscales Perceived Criticism, Critical Comments, Emotional Over-involvement, Expressivity / Spontaneity and Chaos). For details see Table 1.

**Table 1:** Pre (T1 *n* = 82), post (T2 *n* = 48) and follow-up (T3 *n* = 34) results on measures from battery evaluating Family Connections (FC) from family member participants

Variable	Pre-FC (T1)	Post-FC (T2)	FU (T3)	Main effect of time *F* (*df*); *p* *partial η* ^*2*^	Main effect of group *F* (*df*); *p* *partial η* ^*2*^	Time*group Interaction *F* (*df*); *p* *partial η* ^*2*^
	*M (SD)*	*M (SD)*	*M (SD)*			
BSI						
General Severity Index	41.23 (34.39)	31.94 (29.43)	36.44 (27.55)	4.20 (2,23) *p* = 0.028* *η*^*2*^ = 0.268	0.123 (1,24) *p* = 0.729 *η*^*2*^ = 0.005	1.20 (2,23) *p* = 0.319 *η*^*2*^ = 0.094
BAS, total	21.97 (12.45)	15.92 (11.50)	14.44 (8.02)	5.06 (2,17) *p* = 0.019* *η*^*2*^ = 0.373	5.26 (1,18) *p* = 0.034* *η*^*2*^ = 0.226	5.06 (2,17) *p* = 0.017* *η*^*2*^ = 0.373
Activity limitations	9.78 (7.38)	7.21 (6.53)	6.25 (4.72)	3.03 (2,20) *p* = 0.071 *η*^*2*^ = 0.233	6.56 (1,21) *p* = 0.018* *η*^*2*^ = 0.238	0.74 (2,20) *p* = 0.490 *η*^*2*^ = 0.069
Worry and guilt	8.38 (3.51)	6.09 (3.24)	5.44 (2.98)	11.79 (2,21) *p* < 0.001*** *η*^*2*^ = 0.529	0.05 (1,22) *p* = 0.830 *η*^*2*^ = 0.002	6.35 (2,21) *p* = 0.007** *η*^*2*^ = 0.377
Social strain	4.09 (3.41)	2.80 (2.74)	2.88 (2.66)	2.74 (2,21) *p* = 0.108 *η*^*2*^ = 0.207	3.69 (1,22) *p* = 0.068 *η*^*2*^ = 0.045	5.80 (2,21) *p* = 0.010** *η*^*2*^ = 0.356
Composite score for difficult family climate	5.01 (3.57)	3.39 (3.47)	2.25 (2.63)	9.37 (2,17) *p* = 0.002** *η*^*2*^ = 0.524	0.84 (1,18) *p* = 0.373 *η*^*2*^ = 0.157	3.39 (2,17) *p* = 0.058 *η*^*2*^ = 0.285
QAFM						
Perceived Criticism	15.50 (5.52)	13.85 (4.16)	12.94 (3.86)	7.00 (2,21) *p* = 0.005** *η*^*2*^ = 0.400	3.35 (1,22) *p* = 0.081 *η*^*2*^ = 0.132	5.92 (2,21) *p* = 0.009** *η*^*2*^ = 0.361
Perceived emotional involvement	11.99 (2.89)	11.31 (3.00)	11.71 (2.68)	2.16 (2,23) *p* = 0.138 *η*^*2*^ = 0.158	5.77 (1,24) *p* = 0.024* *η*^*2*^ = 0.194	0.424 (2,23) *p* = 0.660 *η*^*2*^ = 0.036
Critical Comments	23.88 (6.91)	21.23 (6.99)	19.84 (6.56)	3.62 (2,19) *p* = 0.046* *η*^*2*^ = 0.276	0.86 (1,20) *p* = 0.364 *η*^*2*^ = 0.041	4.02 (2,19) *p* = 0.035* *η*^*2*^ = 0.297
Emotional Over-involvement	19.21 (4.64)	17.56 (5.01)	16.00 (4.13)	5.68 (2,22) *p* = 0.010* *η*^*2*^ = 0.341	0.254 (1,23) *p* = 0.619 *η*^*2*^ = 0.011	1.71 (2,22) *p* = 0.204 *η*^*2*^ = 0.135
FCS						
Closeness	4.66 (3.71)	5.48 (3.38)	5.71 (3.67)	0.92 (2,23) *p* = 0.414 *η*^*2*^ = 0.074	1.89 (1,24) *p* = 0.182 *η*^*2*^ = 0.073	3.86 (2,23) *p* = 0.036* *η*^*2*^ = 0.251
Distance	0.88 (1.21)	0.46 (1.01)	0.50 (0.86)	0.71 (2,23) *p* = 0.500 *η*^*2*^ = 0.058	1.17 (1,24) *p* = 0.290 *η*^*2*^ = 0.046	1.78 (2,23) *p* = 0.191 *η*^*2*^ = 0.134
Expressivity / Spontaneity	0.70 (0.99)	0.33 (0.48)	0.32 (0.64)	4.83 (2,23) *p* = 0.018* *η*^*2*^ = 0.296	0.656 (1,24) *p* = 0.426 *η*^*2*^ = 0.027	2.58 (2,23) *p* = 0.097 *η*^*2*^ = 0.183
Chaos	1.79 (1.44)	1.35 (1.71)	1.06 (1.35)	3.95 (2,23) *p* = 0.034* *η*^*2*^ = 0.256	0.216 (1,24) *p* = 0.647 *η*^*2*^ = 0.009	1.17 (2,23) *p* = 0.327 *η*^*2*^ = 0.092
KIMS						
Observe	35.49 (8.67)	34.56 (9.65)	37.00 (9.72)	0.07 (2,23) *p* = 0.932 *η*^*2*^ = 0.006	0.56 (1,24) *p* = 0.461 *η*^*2*^ = 0.023	4.14 (2,23) *p* = 0.029* *η*^*2*^ = 0.265
Describe	26.99 (6.41)	27.07 (5.82)	27.84 (6.54)	1.05 (2,22) *p* = 0.368 *η*^*2*^ = 0.095	2.88 (1,23) *p* = 0.103 *η*^*2*^ = 0.111	1.11 (2,22) *p* = 0.349 *η*^*2*^ = 0.100
Act with awareness	29.03 (5.89)	29.04 (5.04)	28.85 (5.89)	0.07 (2,23) *p* = 0.930 *η*^*2*^ = 0.006	0.90 (1,24) *p* = 0.353 *η*^*2*^ = 0.036	0.290 (2,23) *p* = 0.751 *η*^*2*^ = 0.025
Accept without judgement	31.28 (7.31)	33.07 (6.37)	32.25 (7.67)	0.66 (2,22) *p* = 0.527 *η*^*2*^ = 0.062	0.95 (1,23) *p* = 0.340 *η*^*2*^ = 0.040	0.42 (2,22) *p* = 0.665 *η*^*2*^ = 0.40
QOLI	2.45 (1.61)	2.66 (1.36)	2.40 (1.42)	0.19 (2,20) *p* = 0.829 *η*^*2*^ = 0.019	2.61 (1,21) *p* = 0.116 *η*^*2*^ = 0.111	0.34 (2,20) *p* = 0.717 *η*^*2*^ = 0.033

*BSI* Brief Symptom Inventory, *BAS* Burden Assessment Scale, *QAFM* Questions about Family Members, *FCS* The Family Climate Scale, *KIMS* Kentucky Inventory of Mindfulness Skills, *QOLI* Quality of Life Inventory

* *p* < .05; ** *p* < .01; *** *p* < .001

As further shown in Table 1 the Kentucky Inventory of Mindfulness Skills and quality of life as assessed from the Quality of Life Interview did not improve over the course of FC or by follow-up assessment.

## Discussion

Our main findings are that participants who received FC reported lower mental health difficulties, lower perceived burden of caring for a family member with severe mental health difficulties, and higher global family functioning from pre- to post-intervention. These improvements persisted for as long as six-to-7 months following the post-test, independent of the intensity (12 weekly sessions versus two intensive weekends) in which FC was delivered. This is in line with pioneering studies evaluating the effects of FC [13, 15, 21] and subsequent FC evaluation research conducted in Europe [16, 17]. Confirming and extending the findings reported in Lundh and Wångby’s first Swedish evaluation [15] to a context in which two different therapeutic intensities of FC were delivered was an overarching aim of the current study. We therefore have preliminary results suggesting that DBT service providers may usefully administer FC-R in DBT-R settings as a time-and-cost efficient implementation option for doing so.

A hypothesis that our data confirmed was persisting challenges at T2 for the FC-R participants who received a loved one home from residential treatment at the time of the post-FC assessment. Positive change was demonstrated by these participants at T3, indicating that despite the initial stress in the transition of bringing a loved one home from treatment, FC may be effective in helping participants achieve positive change through use of skills acquired over the course of the program. An implication of this finding is that booster sessions around the time of changes in treatment, particularly returning home from residential treatment, may be indicated. FC has been previously tested outside of the population of family members supporting an individual diagnosed with BPD. Drossel, Fisher and Mercer [34] implemented FC for family caregivers of individuals with dementia. These authors observed a need for booster sessions to support gains to family members providing care to a relative with dementia over time, due to the progressive deterioration that characterizes this illness. Similarly, in anticipation of stress, transition or upheaval, a booster FC session or more intensive weekend may provide needed reinforcement and support.

### Expected differences between the FC literature and the current study

Although we expected the majority of our results to be consistent with the existing FC evaluation research, two important differences were expected prior to the inception of this study. One difference concerns the stability of positive change reported by more than two thirds of participants in Lundh and Wångby’s sample [15]. Because living situation may change for family members with a relative participating in FC-R (that is, the family member may be discharged and move home over the course of the study), we expected improvement at all time-points would be more likely in the outpatient (DBT-S) setting only. This was reflected by our second hypothesis, and was confirmed by our results.

Another expected difference was accounting for group differences at baseline between FC-S relatives versus FC-R participants. In this study, loved ones receiving DBT-S met criteria for BPD, exhibited extreme difficulties with emotion regulation, or such persisting self-harm and suicidality that DBT was indicated. Criteria for entry into the DBT-R setting were multiple comorbidities, often including BPD, and longstanding severe psychiatric impairment that precluded employment or independent living. In many cases DBT-R-based individuals have lifetime histories of mental illness including experiences of severe inter-generational mental illness. Little is known about how their relatives respond to FC. Evaluation of the latter is a unique contribution of this study. The fact that FC-R family members also showed improvement in their outcomes gives preliminary support that the FC-R does not lose its capacity to produce positive change.

### Limitations

Although our data replicate a number of positive changes reported in the pioneering American, Swedish and FC literature [13, 15, 21], we did not observe any improvements in mindfulness or quality of life over testing periods. Accordingly, non-significant findings might be related to the sample size in our study, by no means excluding clinically relevant effects in the population. The limited sample size is partially related to participants not completing FC or some of the assessments. While incomplete data may introduce a bias, there was no indication for systematic differences between participants completing versus not completing the treatment and the assessments following FC. However, the findings presented in this paper primarily apply to participants who completed FC.

Importantly, the loved ones diagnosed with BPD in this study all received DBT (either outpatient or residential, but they were not randomized) while their relatives participated in FC. This is both a strength and a limitation. It is a strength because no previous studies of FC have controlled for treatment of the loved one with BPD. It is a weakness because it is difficult to generalize findings from this study to individuals and their families when the individual is in a different treatment, or no treatment at all. The absence of a control group also prevents us from drawing causal conclusions. Further, there were no ratings of DBT quality/adherence, so we cannot be sure whether treatment was better in one condition. However, there is a larger “dose” of DBT in residential treatment.

Because this was a study of family members’ experiences of FC, we did not collect data on the use of pharmacotherapy, changes in psychosocial functioning and life satisfaction in loved ones receiving DBT. In retrospect we believe this is a limitation of the study. Pharmacotherapy may contribute to behaviour change [35], and use of medications are quite common in BPD populations [36]. Life satisfaction and engagement in one’s community are other important aspects of personal recovery [37]. In order to attribute improvement more specifically to FC programming, collecting data on medication use and conducting analyses that control for specific psychopharmacological classes of medications prescribed would have strengthened our findings. Measuring social and vocational functioning as well as life satisfaction in BPD populations are also valuable indicators of change over the course of DBT and thereafter [37].

Finally, the Swedish context in which we conducted our study, where DBT is widely available in publicly-funded settings is somewhat unique internationally. Accordingly, our study may be difficult to replicate with FC delivered by service providers. However, in North America and other English-language locations without wide access to publicly-funded DBT, family members who are FC graduates deliver FC. The National Educational Alliance for Borderline Personality Disorder (NEABPD) provides extensive cost-free online resources for loved ones and professionals, including training and materials to deliver FC. It is currently unknown whether there are differences in FC effectiveness when delivered by service providers compared to family members.

## Conclusions

Participants in our study reported longstanding improvements in core areas of functioning related to their own mental health, their perceived resources to care for their loved one receiving DBT and their overall family functioning, regardless of which FC intensity they received. Some deterioration in these outcomes were observed at T2 for families adjusting to their loved one’s return home from residential treatment, which resolved by T3. Our results demonstrate that FC programming is associated with improvements reported in other FC evaluation research [13, 15–17, 19, 21], even for family members whose loved ones’ severity and chronicity of BPD has necessitated residential treatment. We therefore suggest that FC programming be considered an essential component for treating this population in general, particularly for families with loved ones living with them, planning on living with them or having regular close contact.

Hoffman et al. [13] described “surplus stigma” experienced by families with a member diagnosed with BPD, due to misunderstanding and prejudice towards BPD individuals. These authors note that negative attitudes are prevalent both within society and also within the mental health system. In other words, there is an increased likelihood that individuals with a BPD diagnosis and their families seeking services may be excluded or rejected outside of specialized settings. Anticipating this, it is vital to create as many opportunities and methods for welcoming family members with a loved one receiving DBT to participate in FC while reducing burdens and barriers to participation whenever possible. Herein lies the purpose of our work, which demonstrated positive outcomes of FC offered in differing therapeutic intensities for different populations (FC-R versus FC-S).

Further research is required to replicate these findings, ideally with a larger sample size, a control group, and data collected on use of pharmacotherapy, social and vocational functioning and life satisfaction in the loved one receiving DBT. It would also be helpful for future research to evaluate specifically the impact of booster FC sessions for family members who have completed FC - particularly for families of loved ones completing DBT-R when they return home. Booster sessions may be of additional benefit to family members with loved ones completing outpatient DBT as well, particularly if there are concerns about termination.

### Future directions

Due to the non-experimental, non-randomized design employed by this study, we cannot unequivocally attribute the observed changes to the FC treatment. However, our findings of long-term improvement for FC families on a number of outcomes regardless of treatment setting or intensity of FC delivery warrants consideration of why and how these changes occur. One possibility may be that FC teaches DBT skills to family members, equipping them not only with the same skills taught to their family member in DBT treatment, but also giving family members *a new language* with which to communicate. DBT is a behaviourally-based intervention that uniquely emphasizes the goal of using non-judgmental language that is descriptive rather than interpretational. When attributions for others’ behaviours are ambiguous, DBT (and FC by extension) suggests that those attributions are benign [12, 14]. Out-of-control behaviour is understood to reflect suffering in the absence of the needed skills to regulate emotion. Importantly, family members are also taught emotion regulation skills from DBT, which they might offer to help their family member or use themselves when overwhelmed. In the absence of the negative assumptions inherent in judgmental language, as well as with new DBT skills to replace a sense of helplessness and hopelessness, it may be easier to maintain compassion and a desire to support, rather than to blame or escape. This may be an understandable contrast from how behaviour and intentions are described within the family of a persistently self-harming and suicidal individual if that family is burned out and traumatized. Testing the effects of FC within an experimental study, as well as finding ways of studying changes in the actual family communication to see if this mediates the observed changes, would be a significant contribution to the field.

## Data Availability

The datasets analyzed in the current study are available from the corresponding author on reasonable request.

## Abbreviations

BAS: Burden Assessment Scale
BPD: Borderline personality disorder
BSI: Brief Symptom Inventory
DBT: Dialectical Behaviour Therapy
DBT-R: Residential Dialectical Behaviour Therapy
DBT-S: Standard Dialectical Behaviour Therapy
FC: Family Connections
FC-R: Family Connections, residential delivery (intensified)
FCS: Family Climate Scale
FC-S: Family Connections, standard delivery (weekly for 12–14 weeks)
KIMS: Kentucky Inventory of Mindfulness Skills
QAFM: Questions about the family
QOLI: Quality of Life Inventory
T1: Time 1
T2: Time 2
T3: Time 3

## Footnotes

1 Six-months follow-up was selected for the FC-S comparison. We wanted the FC-R families to have at least a month to complete homework and practice skills taught in the second portion of the FC-R group.
